# Comparison of Cervical Spine Anatomy in Calves, Pigs and Humans

**DOI:** 10.1371/journal.pone.0148610

**Published:** 2016-02-11

**Authors:** Sun-Ren Sheng, Hua-Zi Xu, Yong-Li Wang, Qing-An Zhu, Fang-Min Mao, Yan Lin, Xiang-Yang Wang

**Affiliations:** 1 Department of Orthopedic Surgery, Second Affiliated Hospital of Wenzhou Medical University, Wenzhou, China; 2 Nan Fang Hospital of Southern Medical University, Guangzhou, China; Oklahoma State University, UNITED STATES

## Abstract

**Background Context:**

Animals are commonly used to model the human spine for *in vitro* and *in vivo* experiments. Many studies have investigated similarities and differences between animals and humans in the lumbar and thoracic vertebrae. However, a quantitative anatomic comparison of calf, pig, and human cervical spines has not been reported.

**Purpose:**

To compare fundamental structural similarities and differences in vertebral bodies from the cervical spines of commonly used experimental animal models and humans.

**Study Design:**

Anatomical morphometric analysis was performed on cervical vertebra specimens harvested from humans and two common large animals (i.e., calves and pigs).

**Methods:**

Multiple morphometric parameters were directly measured from cervical spine specimens of twelve pigs, twelve calves and twelve human adult cadavers. The following anatomical parameters were measured: vertebral body width (VBW), vertebral body depth (VBD), vertebral body height (VBH), spinal canal width (SCW), spinal canal depth (SCD), pedicle width (PW), pedicle depth (PD), pedicle inclination (PI), dens width (DW), dens depth (DD), total vertebral width (TVW), and total vertebral depth (TVD).

**Results:**

The atlantoaxial (C1–2) joint in pigs is similar to that in humans and could serve as a human substitute. The pig cervical spine is highly similar to the human cervical spine, except for two large transverse processes in the anterior regions ofC4–C6. The width and depth of the calf odontoid process were larger than those in humans. VBW and VBD of calf cervical vertebrae were larger than those in humans, but the spinal canal was smaller. Calf C7 was relatively similar to human C7, thus, it may be a good substitute.

**Conclusion:**

Pig cervical vertebrae were more suitable human substitutions than calf cervical vertebrae, especially with respect to C1, C2, and C7. The biomechanical properties of nerve vascular anatomy and various segment functions in pig and calf cervical vertebrae must be considered when selecting an animal model for research on the spine.

## Introduction

Due to the potential for infection, the limited supply of human cadavers, and the ethical concerns surrounding the use of human specimens, vertebrae from animal models, such as pigs [1–9], calves [10–19], dogs [20–27], sheep [28–31] and deer [32–34],have been widely used in spine research to replace human vertebrae. Several factors must be considered when choosing a model animal species, including size, cost, disc geometry, cellularity and biomechanics.

Several studies [4,7,15,18,35] have discussed the anatomy and biomechanics of pig and calf vertebrae, particularly in lumbar and thoracic vertebrae. However, a systematic quantitative comparison of anatomical data corresponding to pig, calf, and human cervical vertebrae has not been reported. In the current study, we evaluated the geometries of cervical vertebrae in two animal models and normalized these parameters for comparison against human cervical vertebrae. Through these comparisons, we assessed the appropriateness of utilizing pig and calf cervical vertebrae as human substitutes for in vitro and in vivo experiments.

## Materials and Methods

Twelve one-year-old pig cervical spines (C0-T1) (weight, 60–80 kg) and twelve one-week-old calf cervical spines (C0-T1) (weight, 40–50 kg) were obtained from a local abattoir. Twelve human cervical spines (C0-T1) from adults between30 and 40 years of age (weight, 60–80 kg) were obtained from WenZhou Medical College. A spine specialist evaluated the human vertebrae to ensure that cervical syndrome and osteoporosis were not present. The WenZhou Medical College Ethics Committee reviewed and approved the study and waived the need for informed consent. The cervical spines were stored at -20°C prior to preparation and testing. All musculature and ligaments were carefully removed so that the underlying bony structures were not damaged.

**Fig 1:**
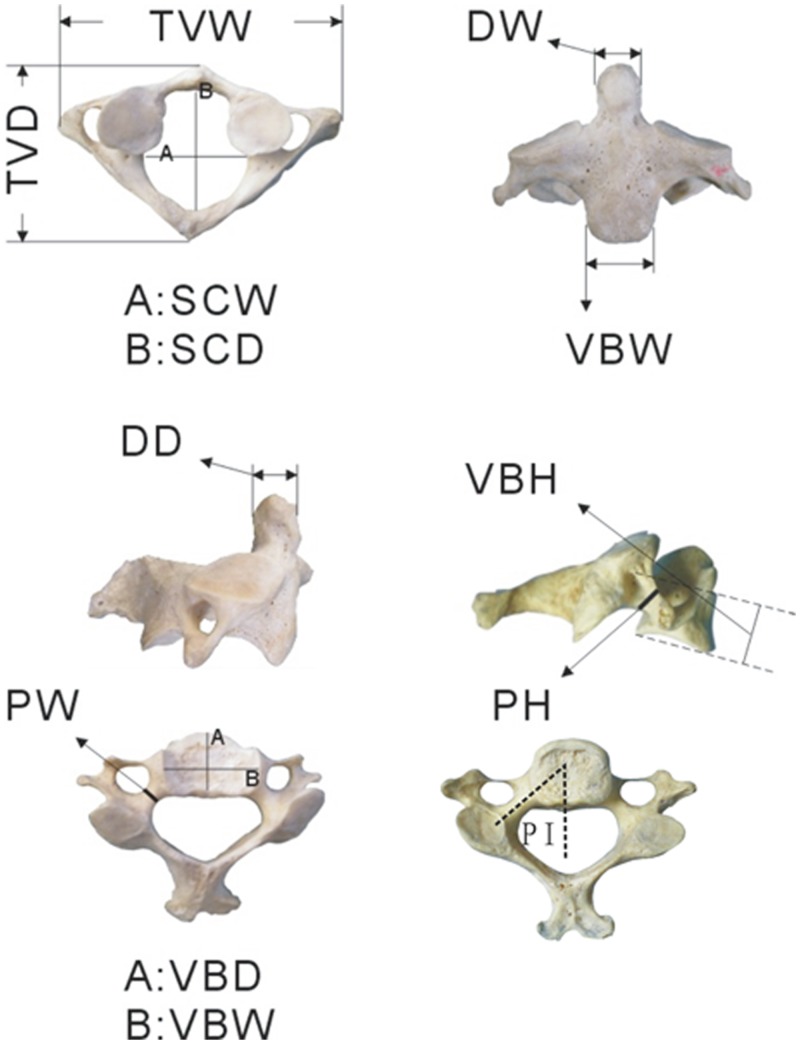
Anatomical parameters. (VBW) vertebral body width, (VBD) vertebral body depth, (VBH) vertebral body height, (SCW) spinal canal width, (SCD) spinal canal depth, (PW) pedicle width, (PH) pedicle height,(PI) pedicle inclination, (DD) dens depth, (DW) dens width, (TVD) total vertebral depth, and (TVW) total vertebral width.

Vernier calipers (Gaozhi_0–200, Shanghai, China; accuracy ±0.03 mm) and a protractor, both meeting international standards, were respectively used to take linear and angular measurements. The following anatomic parameters were measured directly from the surface of the spine specimens: vertebral body width (VBW), vertebral body depth (VBD), vertebral body height (VBH), spinal canal width (SCW), spinal canal depth (SCD), pedicle width (PW), pedicle depth (PD), pedicle inclination (PI), dens width (DW), dens depth (DD), total vertebral width (TVW), and total vertebral depth (TVD) (Table 1, Fig 1). Our sample size was consistent with previous similar studies [30,32,35,36]. Each measurement was repeated three times by two independent observers, and the mean value was recorded. All anatomic values are expressed as the mean ± standard deviation (SD). Differences among the calf, pig, and human spines were statistically analyzed using analysis of variance (ANOVA) followed by Dunnett’s test. P values less than 0.05 were considered significant.

**Table 1:** Anatomical Parameters and Abbreviations.

Abbreviation	Dimension
TVW	Total vertebral width
TVD	Total vertebral depth
VBW	Vertebral body width
VBD	Vertebral body depth
VBH	Vertebral body height
SCW	Spinal canal width
SCD	Spinal canal depth
DW	Dens width
DD	Dens depth
PW	Pedicle width
PI	Pedicle inclination
PH	Pedicle height
A: Anterior P: Posterior	U: Upper l: Lower

## Results

### Upper Cervical Vertebrae

There was no vertebral artery foramen around the atlas vertebra in the calf. Compared to the human atlas vertebra, the PI, DD and DW of the calf vertebra were larger, whereas the VBH and VBW were smaller. The calf atlas vertebra was similar to the human atlas vertebra in SCD and TVD. The odontoid process of the calf vertebra was a unique feature, and its length and width were greater than those of the human odontoid process (p<0.05) (approximately 1.7 and 2 times, respectively).

There were few differences in the atlas vertebral body and spinal canal between humans and pigs, but the pedicles of pigs were thicker than those of humans. The PI was greater in the pig upper cervical vertebrae. The pig cervical DD and DW were similar to those in humans (p>0.05). The pig SCD and SCW were larger than those in humans at C2 (p<0.05) (Table 2).

**Table 2:** The means and standard deviations of various anatomical dimensions of the atlantoaxial (C1-2) joints of calves, pigs and humans.

	C1			C2		
	Humans	Calves	Pigs	Humans	Calves	Pigs
SCD	2.73±0.15	3.07±0.69	2.82±0.15	1.97±0.24	1.86±0.17	1.47±0.23#
SCW	2.61±0.18	3.28±0.09※	2.21±0.22#	2.41±0.11	2.30±0.40	1.82±0.14#
PD	1.12±0.05	2.05±0.17※	1.79±0.13#			
PW	0.55±0.03	1.13±0.30※	1.11±0.07#			
PI	46.66±1.3	67.15±3.2※	63.33±2.6#			
DD				1.10±0.04	3.40±0.16※	1.00±0.04
DW				1.07±0.17	1.75±0.17※	0.95±0.07
TVD	8.49±1.04	7.31±0.69	8.58±0.32			
TVW	1.91±0.10	5.39±1.12	6.06±0.16#			
VBDl				1.56±0.16	2.65±0.23※	1.78±0.06#
VBWl				1.91±0.12	3.96±0.38※	3.33±0.21#

(SCW) spinal canal width, (SCD) spinal canal depth, (PW) pedicle width, (PH) pedicle height, (PI) pedicle inclination, (DD) dens depth, (DW) dens width, (TVD) total vertebral depth, (TVW) total vertebral width, (VBDl) vertebral body depth lower, (VBWl) vertebral body width lower,

^(※)^p<0.05, significant difference between humans and calves;

^(#)^ p<0.05, significant difference between humans and pigs.

### Middle and Lower Cervical Vertebrae

The pedicle angles in the middle and lower cervical vertebrae of calves had nearly the same profiles as those in humans. Compared to humans, the VBW and VBD of calf cervical vertebrae were larger, but the spinal canal was smaller (p<0.05). From C3–C7, the calf PW was 1 cm less than that in humans, whereas the PH was 1 cm larger. Compared to humans, the spinous process and transverse processes of calf vertebrae were shorter and more horizontal. From C3 to C7, the difference between human and calf vertebrae gradually decreased. Calf C7 was similar to human C7 in VBW, PI and PW (p>0.05).

**Fig 2:**
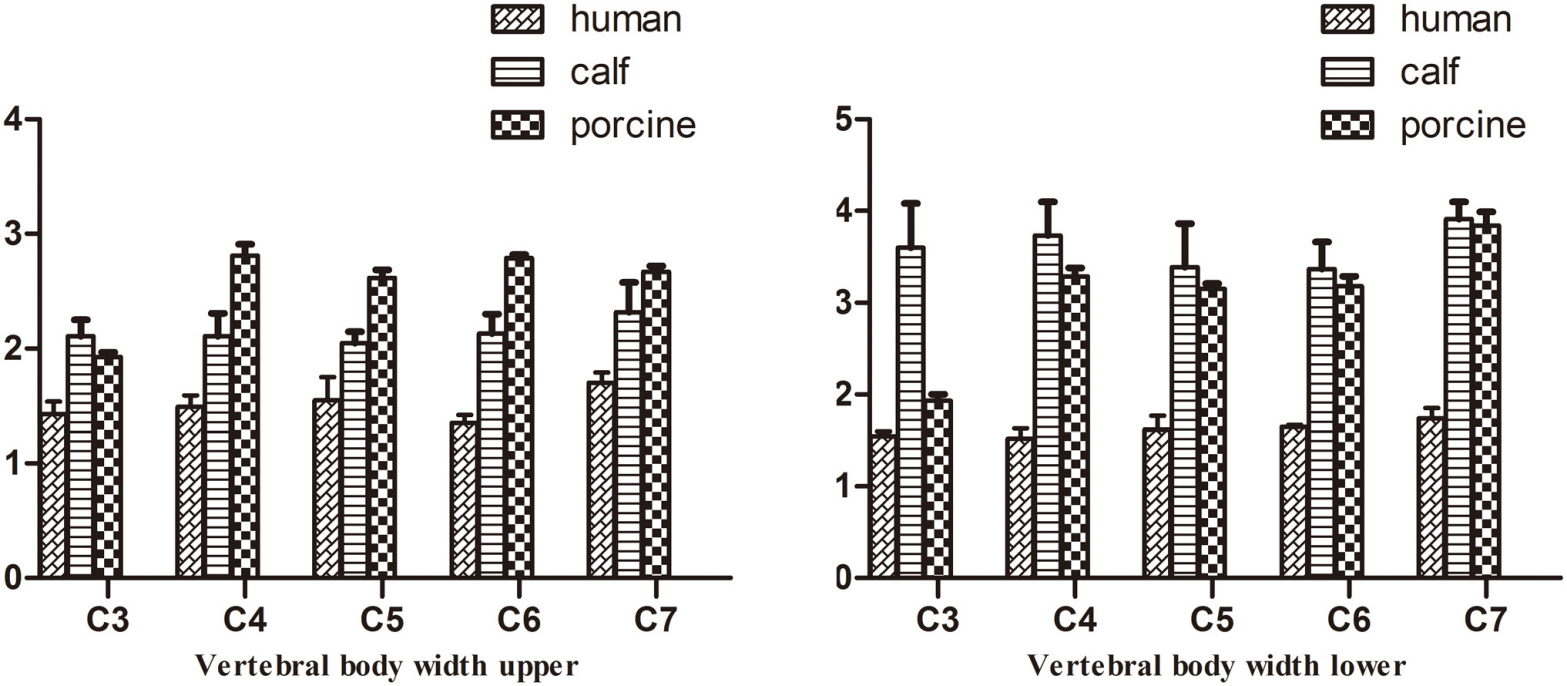
Comparison of vertebral body width (mean ± stand deviation).

**Fig 3:**
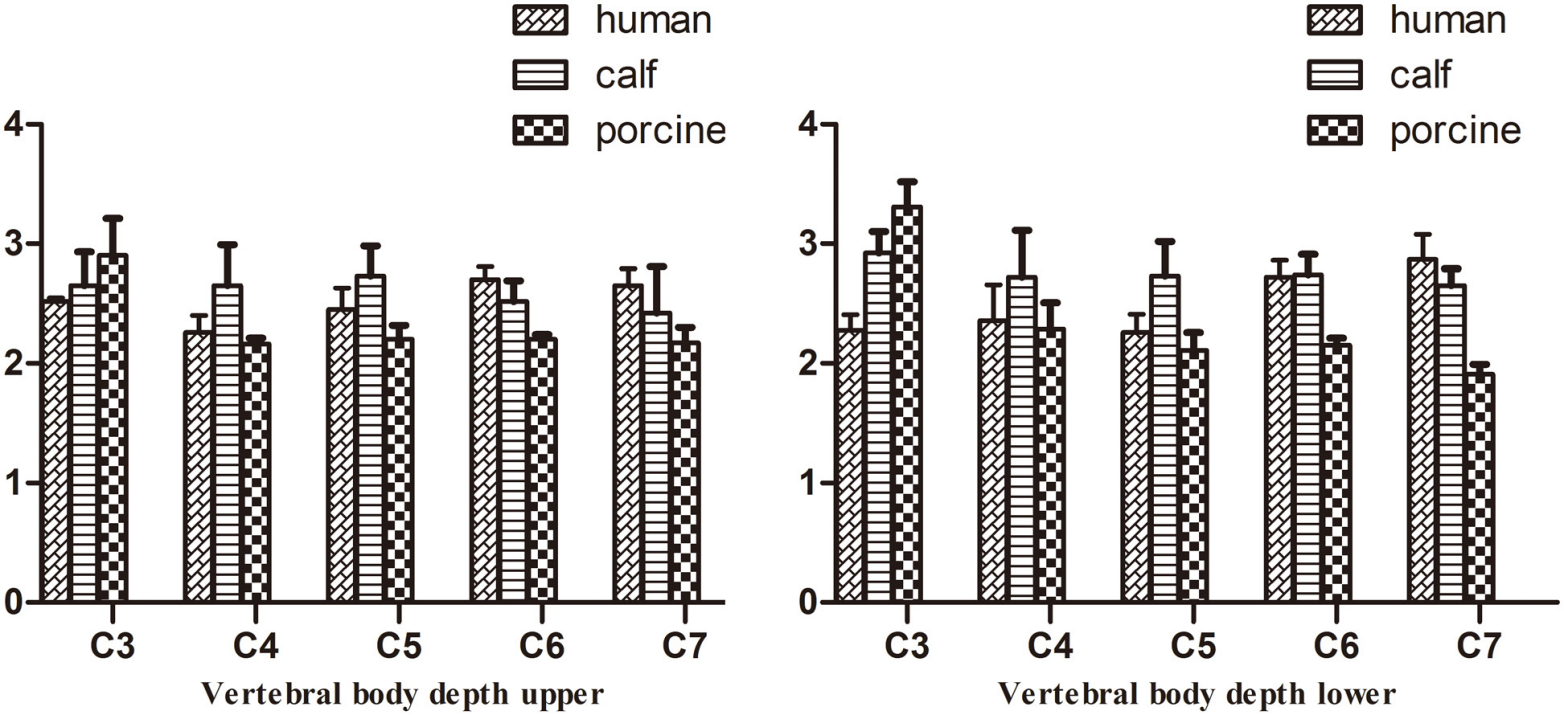
Comparison of vertebral body depth (mean ± stand deviation).

**Fig 4:**
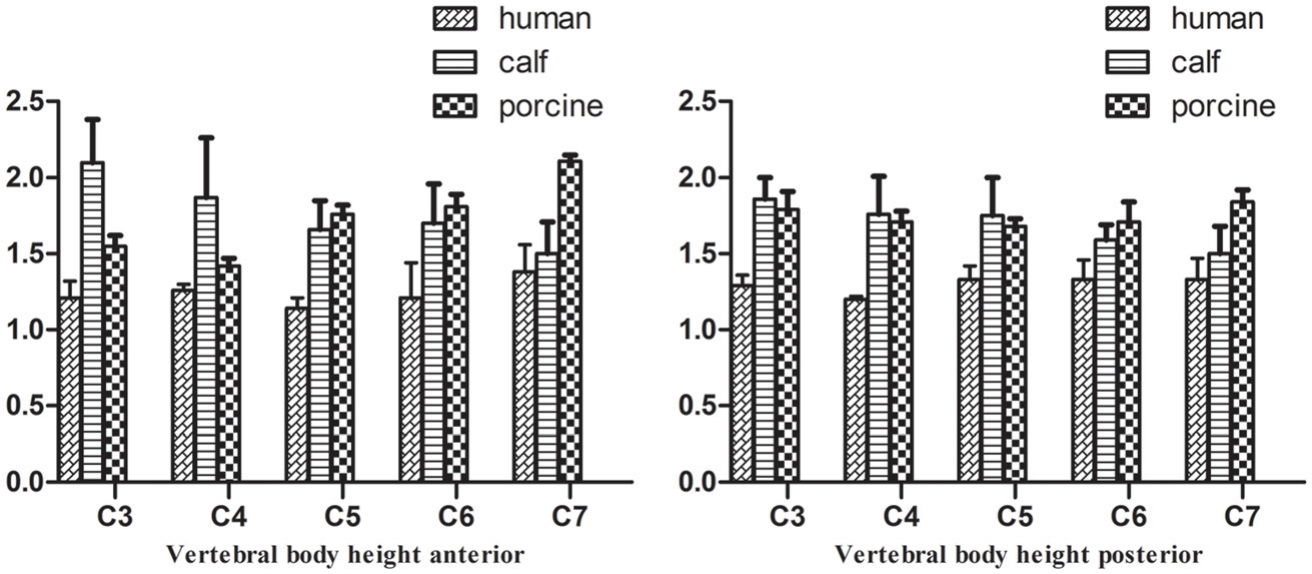
Comparison of vertebral body height (mean ± stand deviation).

**Fig 5:**
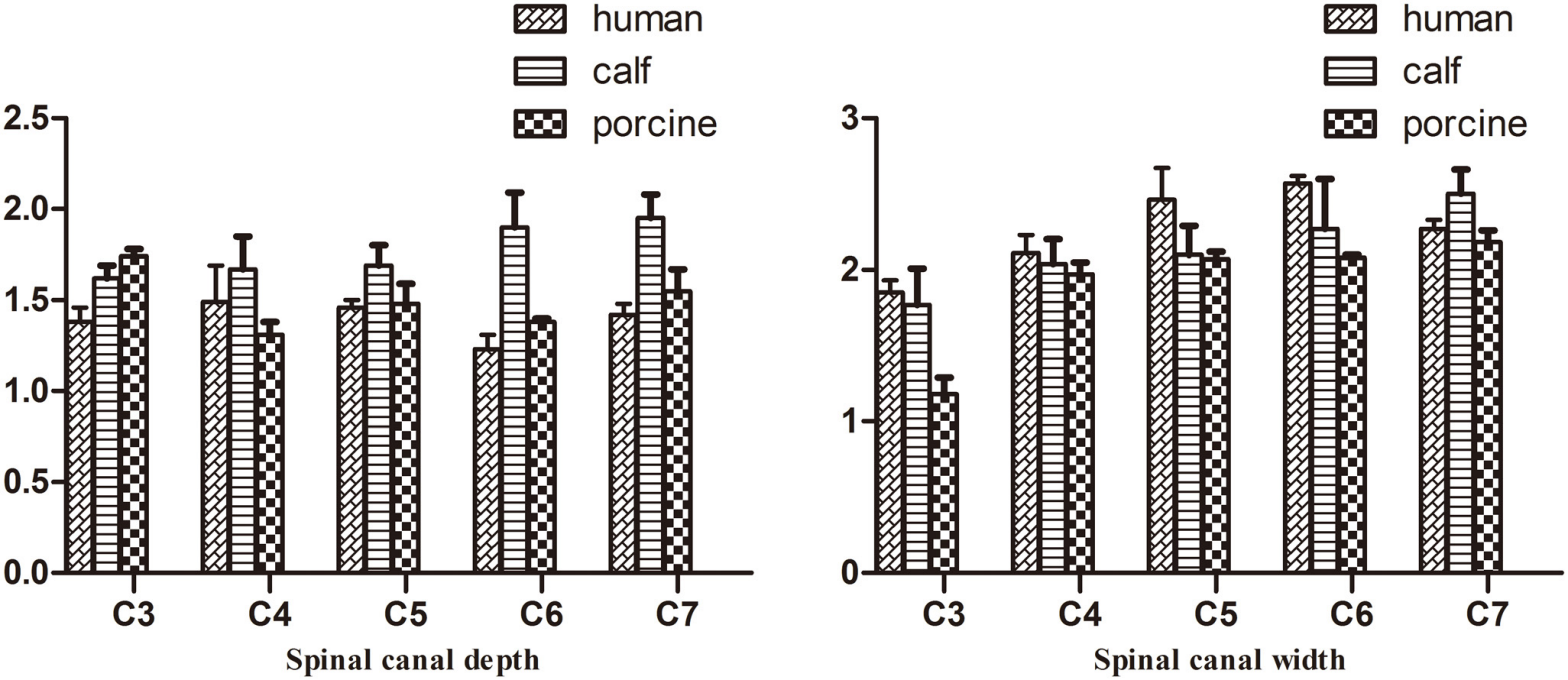
Comparison of spinal canal properties (mean ± stand deviation).

**Fig 6:**
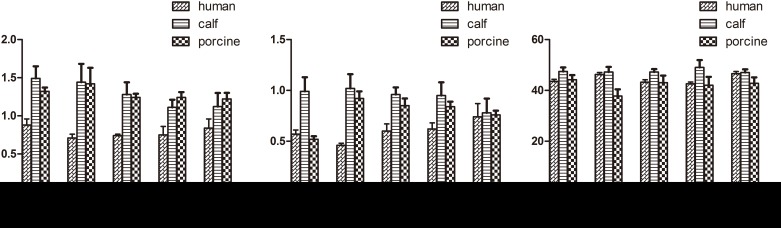
Comparison of pedicle properties (mean ± stand deviation).

Pig cervical vertebrae had larger VBWs than human cervical vertebrae, whereas pig vertebral PI and SCW were nearly the same as those of humans in the middle and lower cervical vertebrae. Similar VBD, VBH, PH, and PW were found between pigs and humans in the middle and lower cervical vertebrae (p>0.05). From C3–C7, the differences in SCD between pig and human cervical vertebrae decreased. There were two large transverse processes in the anterior regions of pig C4–C6 (S1 Table, Figs 2–6). C7 was nearly identical between pigs and humans.

## Discussion

Cervical disease has become widespread due to lifestyle and environmental changes. With the development of internal fixation and surgical methods, an increasing number of spinal surgeons are participating in cervical spine research by testing new implants, spinal fusion techniques, and injury simulations. The majority of current research has utilized specimens derived from large mammals (e.g., sheep [29,30] and baboons [36]) to replace human cervical specimens. Differences exist in the cervical spines of humans and such mammals, although all of the mammals used to date possesss even cervical vertebrae. For example, baboons (*Papio anubis*) [36], which walk upright and are among the closest relatives to humans, have vertebrae with thinner pedicles, longer transverse processes, more prominent uncovertebral joints, and more horizontal spinous processes than humans. However, baboons are extremely rare and not easily obtained for research purposes. Therefore, to identify optimal animal models, we must assess the differences and similarities in the biomechanical properties of animal and human vertebrae. Additionally, we must consider species availability, cost, breeding ability and growth.

Calves and pigs are four-legged mammals with relatively easy-to-obtain cervical spines. In the present study, we evaluated one-year-old pigs weighing 60–80 kg and one-week-old calves weighing 40–50 kg because of their suitable size. Aerssens [37] compared bone composition, density and mechanical competence between humans and animals and suggested that pig bones were the most comparable to human bones. However, Aerssens [37] did not compare differences in morphology. Our current study addressed this deficiency by providing detailed morphological data that can be used in future research.

Calf C1-2 had a different morphology than human C1-2. The calf middle and lower cervical spine were large enough to test new implant systems; however, there were many differences between calf and human vertebrae.

Pig and human cervical vertebrae had similar anatomy, particularly the upper cervical vertebrae. The atlantoaxial joint (C1-2) in pigs was nearly identical to that in humans, particularly with respect to the odontoid process, which could be used to simulate dens fractures and surgical procedures. Cervical vertebrae widths and heights were nearly identical between pigs and humans (phase contrast in 0.1 cm). There was no significant difference in pedicle angle between pig and human cervical vertebrae, while the height and width of the pig pedicle were slightly larger. The pig cervical spine was large enough to test pedicle screws. The largest difference between pig and human middle and lower cervical vertebrae was the presence of two anterior transverse processes in pigs; these were located in the coronal area. The upper and lower vertebral transverse processes were connected, which might affect their biomechanics.

In conclusion, the pig atlantoaxial (C1-2) joint was anatomically identical to the human atlantoaxial joint and could therefore serve as a human substitute. Few differences existed between pig and human vertebrae with respect to the spinal canal, vertebral body, pedicle and articular process. While pig cervical vertebrae were found to have gross similarity to human cervical vertebrae, it must be noted that pigs possess two large transverse processes in the anterior region of the C4–C6 vertebral body. Pig cervical vertebrae were more similar to the human spine than to the calf spine, particularly with respect to C1, C2, and C7.

Several differences existed between humans and calves: 1. Calf cervical vertebrae were approximately 75% larger than those in humans; 2. the calf pedicle was thicker and the pedicle angle larger than those in humans; and 3. the width and depth of the calf odontoid bone were greater than those in humans. Calf C7 was relatively similar to human C7 and therefore may be a good substitute.

This current study is the first to compare cervical vertebrae anatomy between pigs, calves and humans. We present detailed and complete anatomical configuration data for pig and calf cervical spines. When considering these two animal models as human substitutes for in vitro and in vivo experiments, the biomechanical properties of their nerve vascular anatomies and functions across various spinal segments must be taken into account.

## Supporting Information

S1 Table Anatomical dimensions of the middle and lower cervical vertebrae. (DOCX)
