# Supplementary material for: Comparison of Cervical Spine Anatomy in Calves, Pigs and Humans
Source: PLoS One. 2016 Feb 11;11(2):e0148610. doi: 10.1371/journal.pone.0148610 (PMC4750986; doi:10.1371/journal.pone.0148610)
Supplement: S1 Table — (DOCX) [file pone.0148610.s001.docx]

|  |  | SCD | SCW | PH | PW | PI | VBDu | VBDl | VBWu | VBWl | VBHa | VBHp | FH | FW |
| --- | --- | --- | --- | --- | --- | --- | --- | --- | --- | --- | --- | --- | --- | --- |
| C3 | A | 1.38±0.05 | 1.85±0.08 | 0.88±0.08 | 0.57±0.04 | 43.5±0.86 | 2.52±0.03 | 2.28±0.13 | 1.43±0.11 | 1.55±0.05 | 1.21±0.11 | 1.29±0.07 | 1.17±0.04 | 0.75±0.12 |
|  | B | 1.62±0.07^＊^ | 1.77±0.24 | 1.49±0.16^＊^ | 0.99±0.14^＊^ | 47.4±1.62^＊^ | 2.65±0.28 | 2.92±0.18^＊^ | 2.11±0.14^＊^ | 3.60±0.48^＊^ | 2.10±0.28^＊^ | 1.86±0.14^＊^ | 1.71±0.12^＊^ | 1.29±0.13^＊^ |
|  | C | 1.74±0.04^＃^ | 1.18±0.11^＃^ | 1.32±0.05^＃^ | 0.52±0.03 | 44.2±1.83 | 2.90±0.31 | 3.31±0.21^＃^ | 1.93±0.04^＃^ | 1.93±0.07 | 1.55±0.07^＃^ | 1.79±0.12^＃^ | 1.15±0.07 | 1.17±0.06^＃^ |
| C4 | A | 1.49±0.20 | 2.11±0.12 | 0.71±0.05 | 0.46±0.02 | 46.3±0.72 | 2.26±0.14 | 2.36±0.30 | 1.49±0.10 | 1.52±0.11 | 1.26±0.04 | 1.20±0.02 | 1.31±0.13 | 1.10±0.09 |
|  | B | 1.67±0.18 | 2.04±0.16 | 1.44±0.24^＊^ | 1.02±0.14^＊^ | 47.2±2.02 | 2.65±0.34^＊^ | 2.72±0.39^＊^ | 2.11±0.20^＊^ | 3.73±0.37^＊^ | 1.87±0.39^＊^ | 1.76±0.25^＊^ | 1.67±0.13^＊^ | 1.25±0.14 |
|  | C | 1.31±0.07 | 1.97±0.08 | 1.42±0.21^＃^ | 0.92±0.07^＃^ | 37.8±2.64^＃^ | 2.16±0.05 | 2.29±0.22 | 2.81±0.10^＃^ | 3.29±0.09^＃^ | 1.42±0.05 | 1.71±0.07^＃^ | 1.16±0.09 | 1.42±0.04^＃^ |
| C5 | A | 1.46±0.04 | 2.46±0.21 | 0.74±0.02 | 0.60±0.07 | 43.2±0.98 | 2.45±0.18 | 2.26±0.15 | 1.55±0.2 | 1.62±0.15 | 1.14±0.07 | 1.33±0.09 | 1.18±0.06 | 0.89±0.07 |
|  | B | 1.69±0.11^＊^ | 2.1±0.19^＊^ | 1.28±0.16^＊^ | 0.96±0.07^＊^ | 47.2±1.15^＊^ | 2.73±0.25^＊^ | 2.73±0.29^＊^ | 2.05±0.10^＊^ | 3.39±0.47^＊^ | 1.66±0.19^＊^ | 1.75±0.25^＊^ | 1.66±0.12^＊^ | 1.29±0.14^＊^ |
|  | C | 1.48±0.11 | 2.07±0.05^＃^ | 1.24±0.05^＃^ | 0.85±0.07^＃^ | 43.0±2.83 | 2.20±0.12 | 2.11±0.15 | 2.62±0.07^＃^ | 3.15±0.06^＃^ | 1.76±0.06^＃^ | 1.68±0.05^＃^ | 1.35±0.21 | 1.32±0.03^＃^ |
| C6 | A | 1.23±0.08 | 2.57±0.05 | 0.75±0.11 | 0.62±0.06 | 42.6±0.67 | 2.70±0.11 | 2.72±0.14 | 1.35±0.07 | 1.65±0.02 | 1.21±0.23 | 1.33±0.13 | 1.01±0.16 | 0.79±0.13 |
|  | B | 1.90±0.19^＊^ | 2.27±0.33^＊^ | 1.11±0.10^＊^ | 0.95±0.13^＊^ | 49.0±2.94^＊^ | 2.52±0.17 | 2.74±0.17 | 2.13±0.17^＊^ | 3.37±0.29^＊^ | 1.70±0.26^＊^ | 1.59±0.10^＊^ | 1.67±0.18^＊^ | 1.28±0.15^＊^ |
|  | C | 1.38±0.02^＃^ | 2.08±0.02^＃^ | 1.24±0.07^＃^ | 0.84±0.05^＃^ | 42.0±3.35 | 2.20±0.04^＃^ | 2.15±0.06^＃^ | 2.79±0.03^＃^ | 3.18±0.11^＃^ | 1.81±0.08^＃^ | 1.71±0.13^＃^ | 1.49±0.01^＃^ | 1.32±0.08^＃^ |
| C7 | A | 1.42±0.06 | 2.27±0.06 | 0.84±0.12 | 0.74±0.13 | 46.6±0.85 | 2.65±0.14 | 2.87±0.21 | 1.70±0.09 | 1.74±0.11 | 1.38±0.18 | 1.33±0.14 | 1.06±0.19 | 0.90±0.13 |
|  | B | 1.95±0.13^＊^ | 2.50±0.16^＊^ | 1.12±0.18^＊^ | 0.78±0.14 | 47.0±1.26 | 2.42±0.39 | 2.65±0.14 | 2.32±0.26^＊^ | 3.91±0.19^＊^ | 1.50±0.21^＊^ | 1.50±0.18 | 1.59±0.11^＊^ | 1.17±0.15^＊^ |
|  | C | 1.55±0.12 | 2.18±0.08 | 1.22±0.08^＃^ | 0.76±0.04 | 42.8±2.32^＃^ | 2.17±0.13^＃^ | 1.91±0.08^＃^ | 2.67±0.05^＃^ | 3.84±0.15^＃^ | 2.11±0.04 | 1.84±0.08^＃^ | 1.43±0.05^＃^ | 1.10±0.03^＃^ |

S1 Table. Anatomical dimensions of the middle and lower cervical vertebrae

(A)Human, (B) calf, (C) pig.﹡p<0.05, significant difference between humans and calves; ＃ p<0.05, significant difference between humans and pigs.
